# Remnants-preserving ACL reconstruction using direct tendinous graft fixation: a new rat model

**DOI:** 10.1186/s13018-021-02890-9

**Published:** 2022-01-05

**Authors:** Emeline Maurice, Thibault Godineau, Diane Pichard, Hanane El Hafci, Gwennhael Autret, Morad Bensidhoum, Véronique Migonney, Mathieu Manassero, Véronique Viateau

**Affiliations:** 1 Unité de Chirurgie, École Nationale Vétérinaire d’Alfort, Université Paris-Est-Créteil, 7 avenue du Général de Gaulle, 94704 Maisons Alfort Cedex, France; 2 Laboratoire de Biologie, Bioingénierie et Bioimagerie Ostéo-Articulaires (LB30A), UMR 7052, Université Paris Diderot Paris 7, 10 avenue de Verdun, 75010 Paris, France; 3 INSERM, PARCC, Université de Paris, Plateforme Imageries du Vivant, 75006 Paris, France; 4 Laboratoire de Biomatériaux et Polymères de Spécialité (LBPS), Laboratoire Chimie, Structures, Propriétés de Biomatériaux et d’Agents Thérapeutiques (CSPBAT), UMR CNRS 7244, Université Sorbonne Paris Nord, 99 avenue JB Clément, 93430 Villetaneuse, France

**Keywords:** Anterior cruciate ligament reconstruction, Remnants’ preservation, Animal model, Rat, Tendinous autograft, Interference screw, Magnetic resonance imaging

## Abstract

**Background:**

Anterior cruciate ligament (ACL) repair techniques are new emerging strategies prevailing, in selected cases, over standard reconstruction of the ACL with excision of its remnants. Mid-substance ACL tears represent a challenge for ACL repair techniques, and remnants-preserving ACL reconstruction (*rp-*ACLR) using an autograft remains the recommended treatment in this situation. However, morbidity associated with the autograft harvesting prompts the need for alternative surgical strategies based on the use of synthetic scaffolds. Relevant small animal models of mid-substance tears with ACL remnants preservation and reconstruction are necessary to establish the preliminary proof of concept of these new strategies.

**Methods:**

A rat model of *rp-*ACLR using a tendinous autograft after complete mid-substance ACL transection was established. Twelve weeks following surgery, clinical outcomes and knee joints were assessed through visual gait analysis, Lachman tests, thigh perimeter measurements, magnetic resonance imaging, micro-computed tomography, and histology, to evaluate the morbidity of the procedure, accuracy of bone tunnel positioning, ACL remnants fate, osteoarthritis, and autograft bony integration. Results were compared with those obtained with isolated ACL transection without reconstruction and to right non-operated knees.

**Results and discussion:**

Most operated animals were weight-bearing the day following surgery, and no adverse inflammatory reaction has been observed for the whole duration of the study. Autograft fixation with cortical screws provided effective graft anchorage until sacrifice. Healing of the transected ACL was not observed in the animals in which no graft reconstruction was performed. *rp*-ACLR was associated with a reduced degeneration of the ACL remnants (*p* = 0.004) and cartilages (*p* = 0.0437). Joint effusion and synovitis were significantly lower in the reconstructed group compared to the transected ACL group (*p* = 0.004). Most of the bone tunnel apertures were anatomically positioned in the coronal and/or sagittal plane. The most deviated bone tunnel apertures were the tibial ones, located in median less than 1 mm posteriorly to anatomical ACL footprint center.

**Conclusion:**

This study presents a cost-effective, new relevant and objective rat model associated with low morbidity for the preliminary study of bio-implantable materials designed for remnants-preserving ACL surgery after mid-substance ACL tear.

## Background

Anterior cruciate ligament (ACL) rupture is one of the most common sport-related injuries worldwide [1]. Resulting instability of the ACL-deficient knee may lead to significant functional impairment and osteoarthritis [2]. Reconstructive surgery, which is usually performed using a tendinous autograft after entire resection of the ACL remnants [3], has become the advocated treatment of ACL injuries in young active people [4]. Still, morbidity and functional outcomes associated with this technique remain limiting factors, with autograft re-ruptures still occurring in up to 28% in high-risk populations, higher risk for developing moderate to severe osteoarthritis compared to non-surgically treated knees, bone tunnel widening, and persisting knee instability [5–8]. With the increasing interest for regenerative medicine, new emerging strategies focus now on sparing instead of excising the ACL remnants, to promote biological healing, through primary repair combined with augmentation using internal bracing, suture anchors, and synthetic devices, or through remnants’ preservation and/or tensioning, combined with reconstruction using tendon grafts [9]. Remnants-sparing techniques are indeed expected to provide many advantages over standard reconstruction techniques, including improved proprioception, anterior stability, Lysholm score and bone tunnel positioning, restoration of native knee kinematics, reduced tibial tunnel enlargement, and lower incidence of OA [9, 10]. However, ACL repair and augmentation techniques only apply to selected cases with proximal ruptures and good ligamentous tissue quality [10]. Mid-substance ACL tears are the most common type of ACL tear and refer to partial or complete ruptures occurring at 25–75% of distal–proximal length of the ligament [10]; they represent adverse healing conditions mainly pertaining to reduced vascularization at this level [11]. Remnants’ preservation must be associated with reconstruction using an autograft in these challenging situations [10]. The limitations associated with the use of autografts prompt the need for alternative strategies in which bio-implantable materials could substitute to the autograft. To the best of our knowledge, well-characterized and relevant small animal models of mid-substance ACL tear with remnants preservation, to establish proof of concept of these new strategies, are lacking.

Several models of remnants-preserving ACL reconstruction (*rp*-ACLR) have been described in large animal species such as goats, sheep, pigs, and dogs [12–15]. Their major drawbacks pertain to their high cost which precludes routine use in preliminary studies in which screening of different materials or constructs is performed. Small animal models are thus mandatory, because they are often used for preliminary proof-of-concept studies, as they are cost-effective and easy to house, before performing experiments on large animals who carry greater logistical and financial considerations [16]. Among them, rabbits have been used as *rp*-ACLR models [17, 18]. However, because these animals consistently hold their knee in hyper-flexion and are kept in small cages in which ambulation is restricted, they are not the best suitable small animal model for this research field in which postoperative weight-bearing is of paramount importance and requires free, unrestricted ambulation [19]. Rat knees share anatomical similarities with the human knee [20], and these small animals can adopt erect bipedal stance [21]. Moreover, their size allows to perform mechanistical analysis, such as bioluminescence imaging, which is a useful tool in the field of tissue engineering [22]. Many rats models have been developed for ACL reconstructions experiments implying synthetic ligaments [16, 23]; however, most of these models did not preserve the ACL remnants (a complete resection was performed) or used indirect fixation of the graft such as periosteal sutures [24–27]. The only model in which *rp*-ACLR surgery with direct graft to bone fixation was performed used a tendinous allograft instead of autograft, for reconstruction [28]. Moreover, none of these models precisely documented the accuracy of the bone tunnels aperture positioning in the joint regarding ACL tibial and femoral footprints, which is a key issue in ACL surgery since non-anatomically positioned bone tunnels have been implicated in failures of ACL reconstructions [29].

The objectives of the present study were (i) to develop a new, rat model of remnants-preserving ACL reconstruction surgery with a tendinous autograft after ACL mid-substance transection, and (ii) to document the effects of the ACL reconstruction on the fate of ACL remnants compared to an absence of reconstruction following transection of the ACL. The main perspective was to obtain a cost-effective and well-characterized, relevant, reproducible, objective, mid-substance ACL tear small animal model with low morbidity that would be available for further studies on bio-implantable materials designed for *rp*-ACLR.

## Materials and methods

### Animals

Eleven female, Wistar rats (age 27 weeks; median weight 350 g [335; 357]) were obtained commercially from a licensed vendor and raised according to current guidelines established by the European Union (specifically, Directive 2010/63/EU-European Convention ETS 123). All animal procedures were approved by the Scientific Ethical Committee ANSES/ENVA/UPEC–APAFIS reference 2018040312168559–14376. Rats were randomly assigned into two groups: the “Autograft” group (AG group, *n* = 6, positive control group), in which the mid-substance ACL transection was followed by *rp*-ACLR using the flexor digitorum tendon as autograft, and the “Isolated Transection” group (IT group, *n* = 5, negative control group) in which isolated transection of the ACL was performed and not followed by *rp*-ACLR; this latter group served as control non-stabilized group. Each right, unoperated knee of all animals served as normal control.

### Surgical procedure

**Fig. 1:**
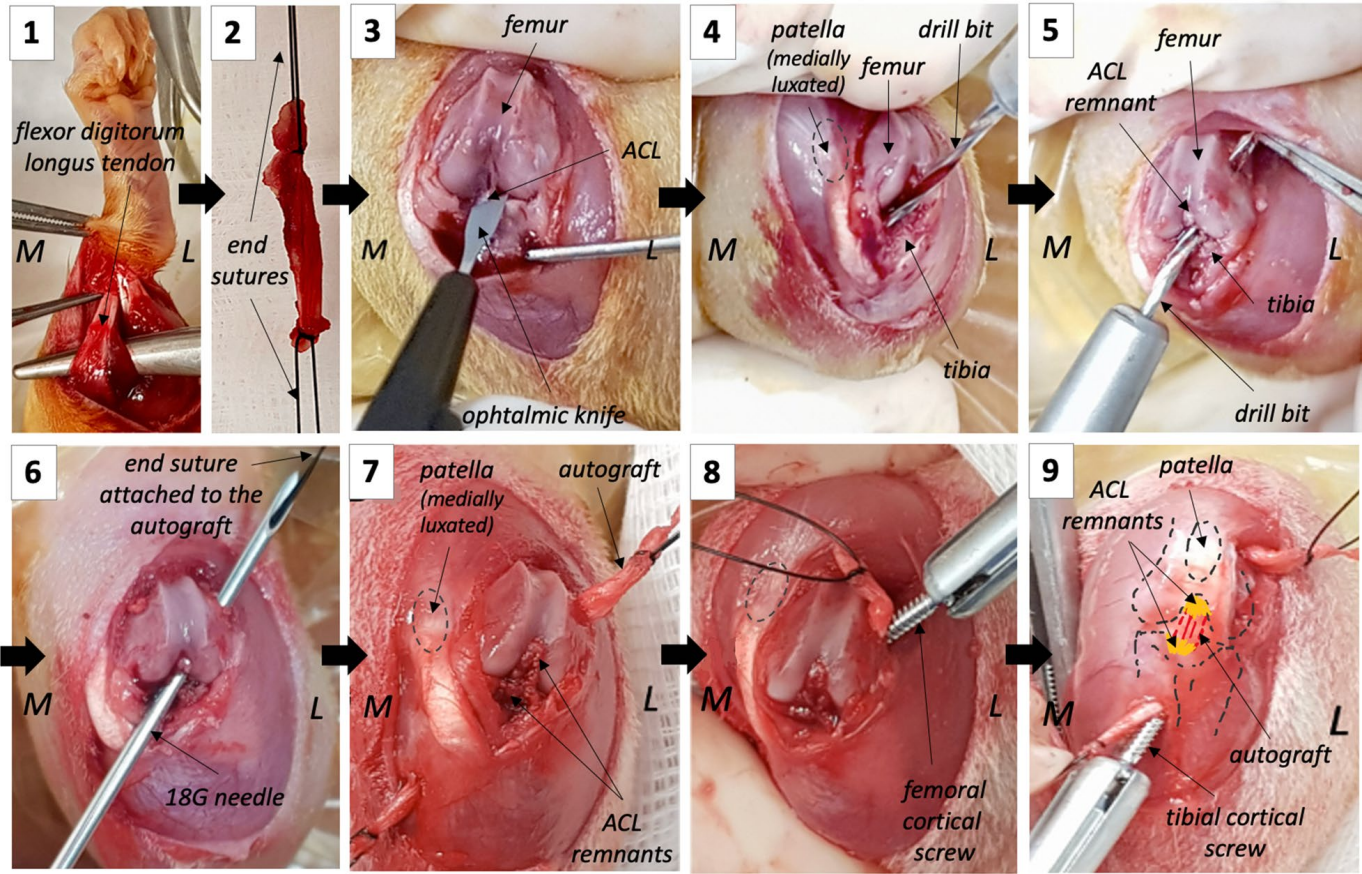
Schematic representation of the surgical procedure. 1. Autograft harvesting. 2. End sutures are placed at both extremities of the autograft. 3. After arthrotomy, the ACL is transected at its mid-substance using an ophthalmic knife. 4. Tibial bone tunnel is drilled from the inside of the joint, starting at the ACL remnant tibial insertion. 5. Femoral bone tunnel is drilled. 6. The end suture is passed through an 18G needle placed into the bone tunnels to serve as a guide. 7. The autograft is in place. 8. The autograft is fixed with a cortical screw on the femoral side. 9. The autograft is fixed with a cortical screw on the tibial side. In the AG group, the surgical procedure includes steps 1 to 9, whereas in the IT group, the surgical procedure is restricted to step 3. M: medial side; L: lateral side

Anesthesia was induced by inhalation of 3% isoflurane with 2L/min oxygen, delivered using an inhalation chamber. Each rat was then injected intraperitoneally with dexmedetomidine (0.2 mg/kg, IP) and ketamine (80 mg/kg, IP); for the duration of surgery, anesthesia was maintained under 2L/min oxygen delivered via flow-by with a nose cone. Buprenorphine (0.1 mg/kg, SC) and meloxicam (1 mg/kg, SC) were administered just after induction of anesthesia for analgesia and anti-inflammatory treatment. The left knee of each animal was prepared aseptically for surgery. For animals in the AG group [Fig. 1], skin and fascia incisions were made caudally from the tarsus to the midpart of the tibia; the flexor digitorum longus ligament was harvested and kept between wet gauze pads until implantation. All rats underwent a lateral arthrotomy, and section of the ACL at its mid-part was performed using an ophthalmic knife (AccuSharp Ophthalmic 22.5° Straight Knife, Accutome™). The presence of an anterior knee instability was validated through Lachman test. Posterior stability of the knee was also assessed at this time point to verify the absence of posterior cruciate ligament lesion. Subsequent intraarticular joint stabilization was performed for rats of the AG group, while knees were closed routinely without stabilization for animals pertaining to the IT group. In the AG group, bone tunnels of 1.5 mm diameter were drilled inside out in the femoral and tibial metaphysis at the locations of the native ACL footprints. End sutures were placed at both extremities of the autograft. One of them was passed through the eye of an 18 gauges needle inserted into the bone tunnels to serve as a guide, while the other one was secured to a Halsted clamp to prevent inadvertent slippage of the autograft when traction was applied to place it into the bone tunnels. Once positioned, the autograft was manually tensioned while secured to bones using two, 6 to 8 mm long 1.5 mm, stainless steel cortical screws (DePuy™) inserted as interference screws, from the outside of the bone tunnels. Isometry and absence of anterior instability were controlled before closure, which was performed using routine procedures. Povidone iodine was applied on the wound immediately after closure of the skin. Postoperative analgesia was provided to all rats through subcutaneous injections of meloxicam (1 mg/kg, SC; once daily for 4 days) and oral buprenorphine (0.1 mg/kg, SC; every 6 h for 72 h). The animals were housed by two, as they were during the acclimation period, and were left free to ambulate without restriction for the duration of the experiments.

### Clinical outcome

Each animal was observed for its appetite and general health status daily and, at weekly intervals, for the presence of lameness until sacrifice at 12 weeks postoperatively. Rats of both groups were reported as “lame” or “not lame” following the orthopedic evaluation. Thigh perimeter of each animal was evaluated preoperatively and at the time of sacrifice to assess the occurrence of amyotrophy consecutively to surgery. Briefly, for each animal, a mean of three measures, performed at the proximal part of the thigh on the operated leg, was extracted using the same scale tape, as described previously [30, 31]. The percentage of amyotrophy occurring on the operated leg between the preoperative period (*T*_0_) and the time of sacrifice (*T*_0+12 weeks_) was expressed using the formula (*T*_0−_*T*_0+12 weeks_) × 100/*T*_0_. A negative value reflected the onset of amyotrophy, whereas a positive one reflected, on the contrary, a muscle gain. Lachman test was performed on both knees at the time of sacrifice to assess the presence of anterior instability and was qualified as “positive” if anterior instability was observed, or “negative” if not.

### Magnetic resonance imaging

Magnetic resonance imaging was performed following sacrifice and after removal of the screws in each animal in order to assess: (i) joint effusion in both operated and non-operated knees in both groups; (ii) the aspect of the implanted autograft and the position of bone tunnels apertures in the joint in the AG group; (iii) the aspect of the native ACL in both groups, as well as the aspect of the posterior cruciate ligament; and (iv) the position of the femoral and tibial ACL footprints in the unoperated contralateral knees from both groups. Acquisitions were performed with a 4.7 Tesla MRI (Bruker BioSpin MRI™ GmbH) and a cryoprobe allowing high resolution. The three-dimensional MRI sequence used was a steady-state gradient echo FISP with a 50 μm isotropic resolution (FOV: 1.5 × 1.5 × 1.28 cm, matrix: 300 × 300 × 256, TR/TE: 20/5 ms, flip angle: 25°, NEX:2, Tacq: 27 min). Reconstitutions of the axial and coronal planes were obtained with the ParaVision® software from the acquired sagittal planes. The autografts and native ACL remnants were evaluated by a single operator on their whole length in the three planes. In the AG group, femoral and tibial bone tunnel apertures positions were evaluated comparatively to corresponding ACL footprints. Anteroposterior position of the center of the bone tunnel aperture was calculated from the anterior border of the tibia and the femur and normalized relatively to the entire anteroposterior length of the corresponding bone on images obtained in the sagittal plane. Lateromedial position of the center of the tibial and femoral bone tunnel apertures was calculated on the images obtained in the coronal plane from the lateral border of the tibial and femur bones, respectively, and normalized relatively to the entire lateromedial length of the corresponding bone. Same calculation was made for the position of the center of the native ACL insertion on the non-operated contralateral knee. Then, for each specimen and each position (anteroposterior and lateromedial), the difference between the bone tunnel aperture position in the joint and the corresponding native ACL footprints position, both calculated relatively to the corresponding length of the bone, was calculated to identify the deviation of the bone tunnel aperture from physiologic ACL footprints anatomic insertion. Bone tunnel positioning was considered “non-anatomical” when the center of the aperture was located outside the lateromedial and anteroposterior corridors defined by the margins of the native ACL footprints, determined on the right contralateral non-operated knees. Bone tunnel positioning was considered “anatomical” when the center of the aperture was located inside those corridors.

### Micro-computed tomography

**Fig. 2:**
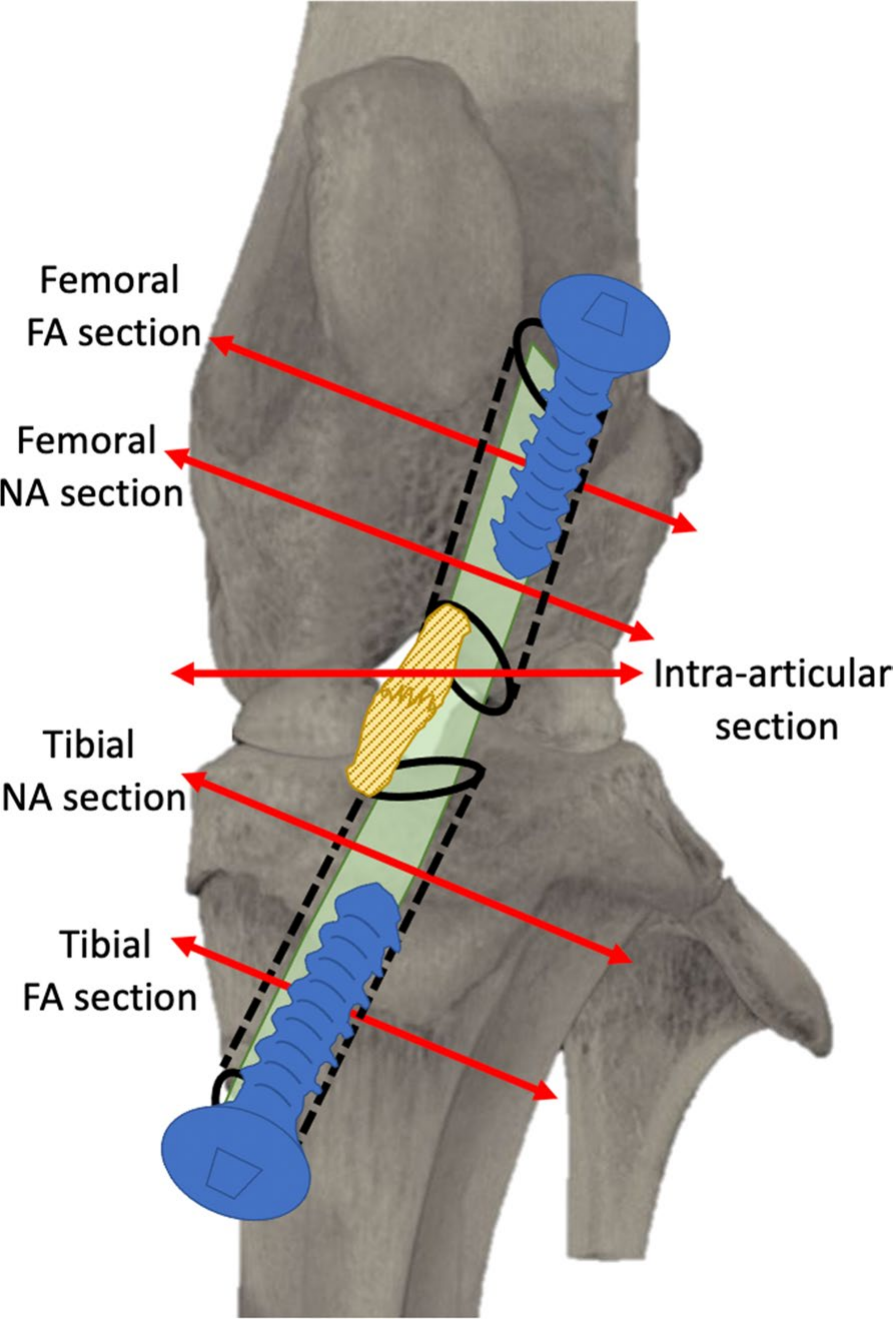
Intra-articular, tibial and femoral sections planes used for micro-CT and histological bone tunnels analysis. The bone tunnels (black dotted lines), autograft (green), ACL remnants (striped yellow), and fixation screws (blue) are represented. Note the direct fixation of the graft provided by the “outside-in” insertion of the cortical screws into the bone tunnels. Screws are represented here for a better understanding, but they were removed before performing the postmortem analysis; thus, only the screw holes were visible on micro-CT and histology sections

Tomodensitometry analysis was performed in all explanted knees (both operated and unoperated contralateral knees) using micro-CT (Skyscan 1172™ MicroCT) before the embedment of each specimen in methyl methacrylate. Data were acquired using Skyscan 1172™ micro-CT Software version 1.5 with constant parameters (80 kV; 100 μA; aluminum filter; duration of exposure 100 ms; resolution 19.98 μm). Reconstitutions were performed using NRecon Skyscan Software™ Ver. 1.7.1.6. The following measurements were performed using CTan Skyscan Software™ ver. 1. They included: (i) bone mineral density and bone microarchitecture (specifically, trabecular separation, thickness, density, and bone volume/total volume), with the patella chosen as the region of interest (ROI); (ii) cross-sectional areas of femoral and tibial bone tunnels for each operated knee of the AG group. These measurements were made in the transverse plane of the tunnels, both at the proximal and distal third of each tunnel and were expressed in µm^2^. Sections made at the proximal third of femoral bone tunnel and at the distal third of tibial bone tunnel were designated “FA sections” (“Far from the Articulation”); sections made at the distal third of femoral bone tunnel and at the proximal third of tibial bone tunnel were designated “NA sections” (“Near the Articulation”) [Fig. 2].

### Non-decalcified histology

All specimens were processed for non-demineralized bone histology. Briefly, the knee joints were fixed using 10% formaldehyde, rinsed using water, dehydrated in ethanol, impregnated in limonene, and embedded in methyl methacrylate. (Screws were removed from the bone tunnels before MRI acquisitions.) The embedded specimens were sectioned in a transverse manner using a circular water-cooled diamond saw (Leitz 1600, Leica™ Inst., Nussloch, Germany). The cutting plan was obtained perpendicularly to the longitudinal axis of the bone tunnels. One section of 500 μm thick was selected from the femoral and tibial tunnels each, located at the proximal and distal third of the bone tunnels. These sections were designated as “NA section” for the section located near the articulation, and “FA section” for the section located far from the articulation [Fig. 2]. Each section was grounded down to a thickness of 100 μm (Mecapol P301, Presi™, Grenoble, France), polished down to 60 μm (Exakt, Apparatebau GmbH & Co KG™, Norderstedt, Germany) and, subsequently, stained using Stevenel’s blue (which stains cellular nuclei in blue) and van Gieson picrofuchsin (which stains bone tissue in pink), following standard procedures. The histology of all specimens was evaluated using normal light.

**Measurement of the synovial membranes thickness** at 12 weeks was based on a previously described method [32]. Briefly, three sections were evaluated per knee, on which three measurements per section were performed on a transverse section of the parapatellar articular capsule located toward the lateral ridge of the femoral trochlea. In total, 9 values per knee were obtained, the average of which served as single value per knee. Results were expressed in µm.

**Osseointegration of the autograft** was evaluated on the entire NA and FA sections of the tibial bone tunnels using a new modification of a previously established semiquantitative graft-to-bone 0–3 scale scoring system [Table 1], based on the fibrous interface width generated between the autograft and the surrounding bone, the bony ingrowth within the autograft, the cell density, the presence of giant cells, and the angiogenesis [33–35]. A score of 15 represented the highest degree of autograft osseointegration according to this scoring system.

**Table 1:** The modified graft-to-bone histologic score

Scoring criteria	0	1	2	3
Fibrous interface width	Wide	Medium	Narrow	Direct contact with bone (no fibrous interface)
Bony ingrowth	None	Mild	Moderate	Marked
Cell density	Marked	Moderate	Mild	Minimal
Giant cells	Lots of giant cells (> 10)	Giant cells moderately present (5–10)	Few giant cells (< 5)	No giant cell
Angiogenesis	Minimal	On a localized part of the periphery	On the entire periphery	On periphery and center

Intra-articular transverse sections were graded histologically for **ACL degenerative changes** according to a previously described ACL degeneration score system [36, 37] based on the following categories: inflammation in the ACL sheath and substance, mucoid degeneration, chondroid metaplasia, cystic changes, and orientation of collagen fibers. Each category was scored on a 0–3 scale where 0 = no changes, 0.5 = minimal changes, 1 = mild changes, 2 = moderate changes, and 3 = severe changes. The highest possible score for ACL degeneration was 15. The ACL was considered normal if total ACL score was 0, mildly degenerative if 0.5–5, moderately degenerative if 5.5–10, or severely degenerative if > 10. Calcium deposition was also assessed, independently of the ACL score.

Graft-to-bone and ACL degeneration scoring were both performed by two investigators. The final score represents the mean value of these two investigators.

### Multimodal evaluation of osteoarthritis

Severity of osteoarthritis at 12 weeks was evaluated by a single operator using a previously established multimodal scoring system developed by the OARSI [38] (Rat Arthritis Knee Scoring System, RAKSS) and combining data obtained from micro-CT, MRI, and histology. Seven primary features of osteoarthritis were measured through this scoring system, namely osteophytes, subchondral sclerosis, joint effusion, bony cysts, bone marrow lesions, loose bodies, and cartilage degeneration (the latter being directly based on modified Mankin’s scoring system [39, 40]). Specifically, Mankin’s score was evaluated on the posterior cartilages of the lateral and medial femoral condyles and scoring system was adapted to assess cartilage integrity on Stevenel’s blue-stained histology sections instead of Safranin-O. A score of 64 represented the maximal degree of severity of knee osteoarthritis according to this 0–64 scale scoring system.

### Statistical analysis

Median and interquartile ranges were used to describe the data. Differences between groups were assessed by unpaired nonparametric *t* test (Mann–Whitney). Correlations between bone tunnel mispositioning and various variables (presence of anterior instability, lameness, graft rupture, bone tunnel area, ACL degeneration, OARSI score) were analyzed using Pearson correlation. Null hypotheses of no difference were rejected if p values were less than 0.05.

## Results

### Clinical outcomes

One rat of the AG group did not recover from anesthesia for reasons unrelated to the surgical procedure. Corresponding explanted knee specimens were kept for the rest of the study to serve as T_0_ specimens. Except for this animal, all rats remained in good health postoperatively, for the whole duration of the study.

Weight-bearing on the operated limb occurred as early as within 24 h after surgery, in all but one animal belonging to the IT group, in which weight-bearing was intermittent at that time point and became permanent the day after. Then, weekly postoperative visual analysis of gait provided evidence of full weight-bearing in all animals until the day of sacrifice. At 12 weeks, none of the animals in the AG and IT groups exhibited marked lameness.

Rats belonging to the AG group exhibited various degrees of digits hyperextension on the operated limb that progressively resolved spontaneously over the month following surgery. In the same group, swelling at the autograft harvesting site was observed during the first days following surgery and disappeared progressively. No adverse local inflammatory response was observed during the following weeks, neither at the harvesting site (for the AG group) nor at the arthrotomy site (in both groups).

At 12 weeks, one specimen of the AG group had positive Lachman test on the operated knee, as well as two specimens of the IT group.

Between *T*_0_ and *T*_0+12 weeks_, the thigh perimeter varied of + 1.5% [− 2.3; 5.3] in the AG group versus − 8.3% [− 10.0; − 1.7] in the IT group (*p* = 0.0159).

### Intra-articular findings

#### MRI evaluation of the AG and ACL remnants

**Fig. 3:**
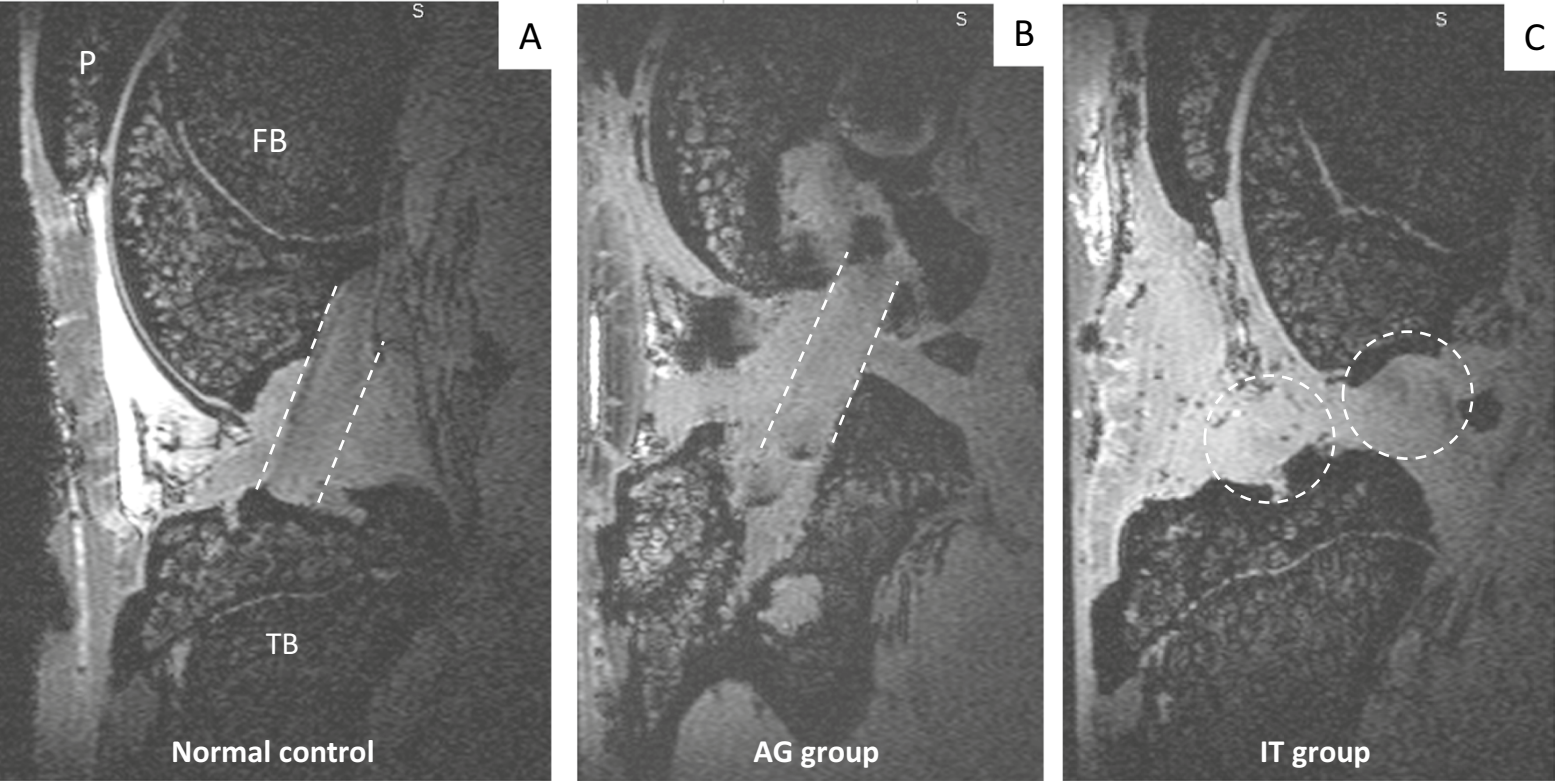
MRI sagittal views of the ACL at 12 weeks. **A** MRI sagittal view of the ACL in a right non-operated knee (dotted lines), **B** MRI sagittal view of the AG and native ACL (dotted lines) in a specimen belonging to the AG group and **C** MRI sagittal view of the non-bridging ACL stumps (dotted circles) resulting from the absence of stabilization of the knee following ACL transection in a specimen of the IT group. P: patella; FB: femur bone; TB: tibial bone

At 12 weeks, continuous ligamentous fibers were observed spanning from the tibia to the femur ACL footprints, in all the specimens of the AG group [Fig. 3B]. These fibers could pertain either to the ACL, to the autograft, or to both. In one animal of the AG group, a partial rupture of the autograft was observed. In the IT group, some fibers pertaining to the ACL remnants stumps were visualized, gathered at the femoral and tibial footprints of the native ACL, but no continuous ligamentous tissue bridging the gap between the ACL stumps was observed in none of the specimens [Fig. 3C]. In each group, right contralateral ACLs appeared normal in all specimens [Fig. 3A]. No posterior cruciate ligament rupture was assessed on MRI examination, in any specimen.

#### MRI evaluation of bone tunnel aperture position

**Fig. 4:**
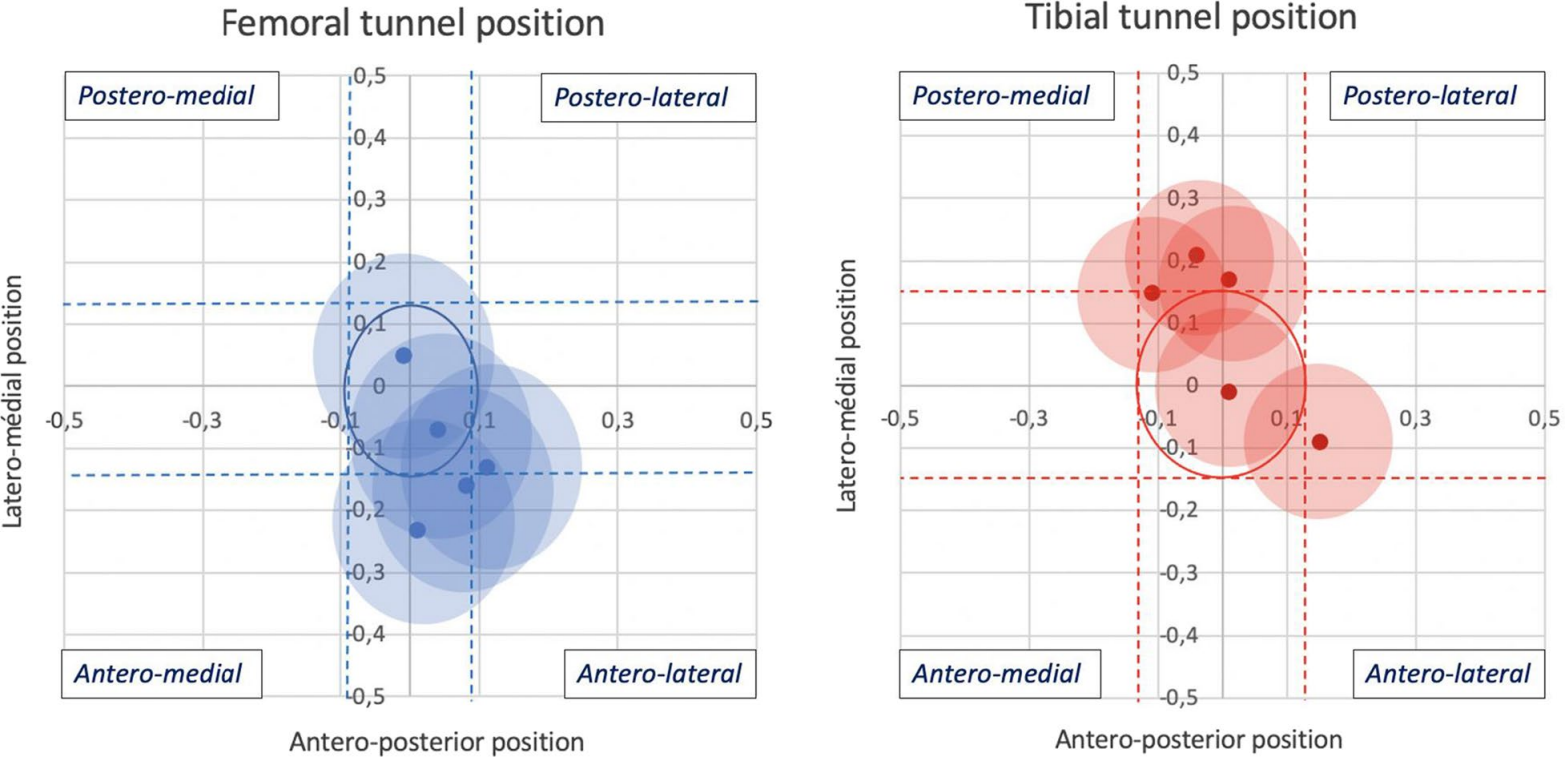
Bone tunnel aperture centers deviations from the center of native ACL footprints. Femoral and tibial bone tunnel apertures positions are represented in the sagittal and coronal planes relatively to the anatomic center of the ACL femoral and tibial footprints, which is represented by the origin of the *X*- and *Y*-axis (*x* = *y* = 0) of both graphs. Ratios between the position of the bone tunnel center relatively to the total length of the bone in each plane were calculated. The same was made for native ACL center. Axis scales correspond to the difference between those two ratios. *X*-axis represents the lateromedial deviation of the bone tunnel aperture from the position of the center of the ACL footprint, relatively to the lateromedial length of the bone; positive values correspond to lateral deviation, while negative values to medial deviation. *Y*-axis represents the anteroposterior deviation of the bone tunnel aperture from the position of the center of the ACL footprint, relatively to the length of the bone measured in the coronal plane; positive values correspond to posterior deviation, while negative values to anterior deviation. Each point designates the center of the bone tunnel aperture of one animal, and each color-filled disc represents the entire bone tunnel aperture area relatively to the corresponding lateromedial and anteroposterior length of the bone. The blue and red circles represent the entire insertion area of the ACL on the femur and tibia, respectively, relatively to the corresponding lateromedial and anteroposterior length of corresponding bones. The dotted lines represent the corridors defined by the margins of the ACL footprints to consider as anatomically or non-anatomically positioned the bone tunnel aperture centers

The center of the femoral bone tunnel apertures was positioned in median 4% [0; 9.5] laterally and − 13% [1; − 19] anteriorly to the center of the ACL femoral footprint. The center of the tibial bone tunnel apertures was positioned in median − 1% [− 7; 8] medially and 15% [− 5; 19] posteriorly to the center of the ACL tibial footprint. We considered as non-anatomically positioned all bone tunnel apertures whose center was located outside the anteroposterior and lateromedial margins of the ACL footprints [see Fig. 4]. Thus, 8/10 bone tunnels were considered anatomically positioned in the coronal plane as well as 6/10 in the sagittal plane; 2 femoral tunnels were too anterior, 1 was too lateral, 3 tibial tunnels were too posterior, and 1 was too lateral. All the variables examined (presence of positive Lachman test, presence of lameness, graft rupture, bone tunnel area, ACL degeneration, OARSI score) did not correlate with the importance of bone tunnel deviation from ACL footprint center. The only animal in which a partial rupture of the graft was strongly suspected was also the only one to have a too laterally positioned tibial bone tunnel aperture.

#### Histological evaluation of ACL degeneration

**Fig. 5:**
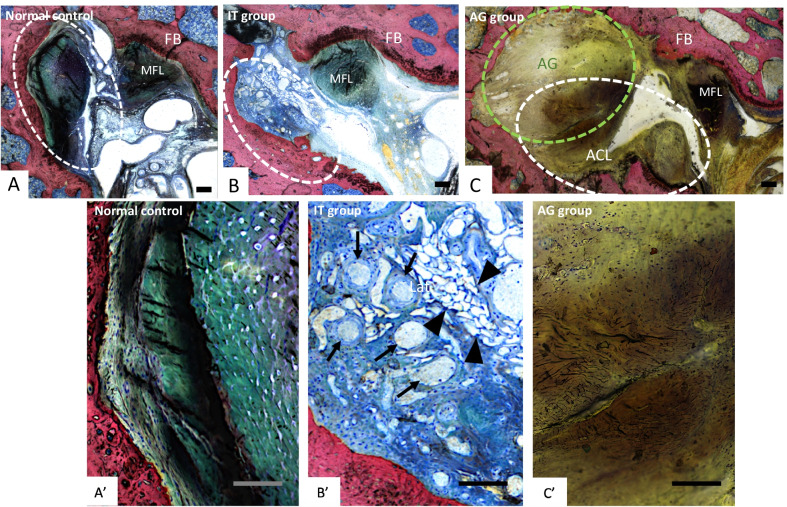
Representative, transverse histological sections of the intraarticular compartment at 12 weeks. **A** Representative transverse histological section of a normal ACL from a right non-operated knee and **A’** its magnification, **B** representative transverse histological section of a severely degenerative ACL from the left operated knee of a specimen belonging to the IT group and **B’** its magnification; note the increased angiogenesis (arrows), cystic changes (arrowheads), and general disorganization of collagen fibers in this IT specimen. **C** Transverse histological section of a mildly degenerative ACL coming from the left knee of a specimen pertaining to the AG group and **C’** its magnification. MFL: menisco-femoral ligament; AG: autograft; ACL: anterior cruciate ligament; FB: femoral bone. Stevenel’s blue and picrofuchsin staining. Bone, cells, and autograft stained pink, blue, and brown, respectively. Scale bars: 200 µm

All the right non-operated knees were given total ACL degeneration score of 0, corresponding to a normal ACL. ACL degeneration scores were significantly higher in the IT group compared to the AG group (10.3 [8.3; 12.4] and 4.5 [3.6; 5.6], respectively, *p* = 0.004) [Fig. 5]. ACL degeneration was classified as “severe” for the IT group and “mild” for the AG group.

#### Histological evaluation of synovial membranes thickness

Synovial membranes of the left operated knees were thicker in the IT group compared to the AG group at 12 weeks (respectively, 418.0 µm [395.5; 448.5] and 347.0 µm [326.5; 395.0], *p* = 0.0278). Synovial membranes thickness of the right non-operated knees was not significantly different between the IT and AG groups (respectively, 158.0 µm [144.5; 178.5] and 153.0 µm [135.5; 164.5], *p* = 0.34). Both groups confounded, synovial membranes of the left operated knees were thicker than those of right non-operated knees (respectively, 395.5 µm [344.5; 434.8] and 155.5 µm [142.3; 167.5], *p* < 0.0001) [**Table ****2**].

**Table 2:** Clinical, micro-CT, and histological results obtained in both groups at 12 weeks

	AG group	IT group	Right knees	*P * value AG versus IT
Thigh perimeter variation (*T*_0+12 weeks_ − *T*_0)_*100/*T*_0_ (%)	1.4 [− 2.3; 5.3]	− 8.3 [− 10.0; − 1.7]	–	0.0159*
Synovial membranes thickness (µm)	347.0 [326.5; 395.0]	418.0 [395.5; 448.5]	155.5 [142.3; 167.5]^a^	0.0278*
ACL degeneration score (/15)	4.5 [3.6; 5.6]	10.3 [8.3; 12.4]	0	0.004*
Inflammation	0.5 [0.1; 1.6]	2.3 [2.0; 3.0]	0	0.0238*
Mucoid degeneration	0.8 [0.4; 1.3]	2.0 [1.1; 2.4]	0	0.0198*
Chondroid metaplasia	1.0 [0.6; 1.8]	2.5 [1.9; 2.8]	0	0.0119*
Cystic changes	0 [0; 0.3]	0.8 [0.6; 2.8]	0	0.004*
Collagen fibers orientation	1.8 [1.4; 2.3]	2.0 [1.6; 2.9]	0	0.2063
Bone mineral density and microarchitecture				
BMD_Left/Right_	0.939 [0.899; 0.951]	0.928 [0.901; 0.951]		0.2738
Trabecular thickness_Left/Right_	1.008 [0.869; 1.048]	0.992 [0.904; 1.008]		0.2738
Trabecular separation_Left/Right_	0.9886 [0.8395; 1.037]	0.9775 [0.9176; 1.033]	_	0.5
Trabecular number_Left/Right_	1.019 [0.965; 1.114]	1.023 [1.006; 1.057]		0.5
Bone volume/total volume_Left/Right_	0.978 [0.945; 1.060]	0.996 [0.956; 1.024]		0.3452
RAKSS score (/64)	21 [16.1; 24.8]	23.3 [19.3; 28.8]		0.2103
Micro-CT-evaluated parameters	17 [12; 21]	14 [13; 20]	6 [5.0; 8.6]^a^	0.4683
MRI-evaluated parameters	3 [2.5; 3]	4 [4; 4]	–	0.0040*
Mankin’s score	1.3 [0.6; 1.6]	3.8 [1.6; 6.1]	0.3 [0.0; 3.4]^a^	0.0437*

^a^Values of the AG and IT groups were combined to obtain these final data when no statistical difference was observed between both groups

*Statistically significant

#### Multimodal evaluation of osteoarthritis

RAKSS score was not significantly different between the AG and the IT groups at 12 weeks [Table 2]. However, joint effusion, which is one of the MRI-evaluated features included in RAKSS score, was significantly lower in the AG group compared to the IT group (3 [2.5; 3] versus 4 [4; 4], respectively, *p* = 0.0040). Mankin’s scores were also significantly lower in the AG compared to the IT group (1.3 [0.6; 1.6] versus 3.8 [1.6; 6.1], respectively, *p* = 0.0437). In each group, Mankin’s scores were not statistically different between the medial and the lateral femoral condyle (*data not shown*, *p* = 0.3452 in the AG group, *p* = 0.3294 in the IT group). Mankin’s scores of the right knees were not statistically different between the IT and AG groups, and the median score for right knees of both groups confounded was 0.3 [0.0; 3.4].

### Intra-osseous findings

#### Micro-CT measurement of BMD and bone microarchitecture

**Fig. 6:**
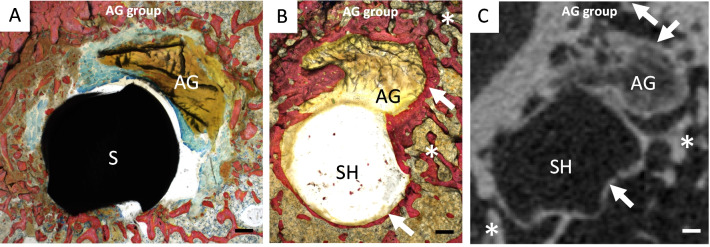
Histology and micro-CT appearance of FA sections from AG specimens at 12 weeks. **A** T0 specimen, **B** representative AG specimen at 12 weeks, and **C** corresponding micro-CT FA section at 12 weeks. Note the cortical (arrows) and cancellous (stars) bone formation surrounding the screw hole (SH) and the autograft (AG) at 12 weeks. The T0 specimen FA section exhibits a transversally cut screw because it was not removed since MRI was not performed for this animal. **A**, **B** Stevenel’s blue and picrofuchsin staining. Bone, cells, and autograft stained pink, blue, and brown, respectively. Scale bars: 200 µm

There were no statistical differences between the AG and IT groups for BMD_Left patella_/BMD_Right patella_, trabecular thickness_Left patella/Right patella_, trabecular separation_Left patella/Right patella_, trabecular number_Left patella/Right patella_, and bone volume/total volume_Left patella/Right patella_ ratios at 12 weeks [Table 2]. Compared with the *T*_0_ specimen, cortico-cancellous bone formation was observed at the direct margins of all bone tunnels and screw hole at 12 weeks [Fig. 6].

#### Micro-CT measurement of bone tunnel areas

When studied independently of their femoral of tibial origin, NA cross-sectional areas tended to be widest than FA cross-sectional areas at 12 weeks (2638 µm^2^ [1547; 3537] versus 1675 µm^2^ [1341; 2121], *p* = 0.0615) [Table 2]. However, at the same time point, there were no statistical differences between median NA and FA tibial cross-sectional areas and between median NA and FA femoral cross-sectional areas.

#### Histological evaluation of graft-to-bone scores

**Fig. 7:**
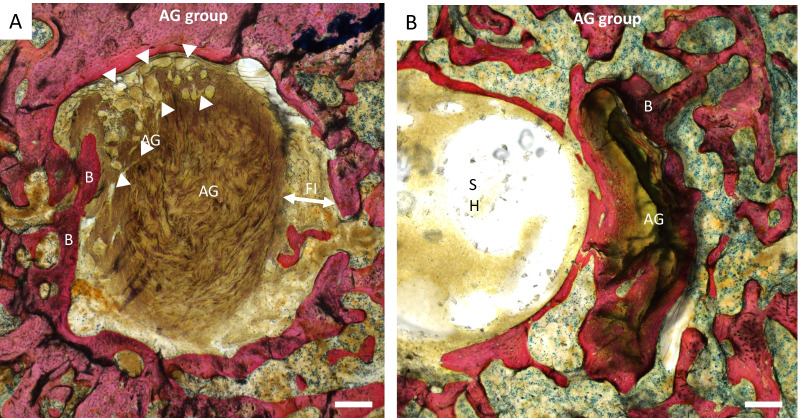
NA and FA tibial histological sections of a specimen implanted with the autograft. **A** NA section: The fibrous interface (FI, bi-headed arrow) is visible between the autograft (AG) and the bone (B). Also note the peripheral neovascularization area (arrowheads). **B** FA section: Direct graft-to-bone contact is visible without fibrous tissue interface. Note the absence of visible vessels and the low cellularity within the autograft. SH: screw hole. Both images show cortico-cancellous bone formation surrounding the autograft and screw hole. Stevenel’s blue and picrofuchsin staining. Bone, cells, and autograft stained pink, blue, and brown, respectively. Scale bars: 200 µm

At 12 weeks, when comparing with the T0 specimen, all examined sections displayed cortico-cancellous bone formation surrounding the autograft and screw hole [Fig. 6]. Graft-to-bone scores were significantly higher in the NA than in the FA sections of the tibial bone tunnel (9.3 [8.5; 12] versus 7.3 [4; 7.5], respectively, *p* = 0.0040) [Table 3 and Fig. 7]. Specifically, cellularity score was significantly increased in the FA compared to NA tibial sections (2 [1.9; 2.5] versus 1.3 [0.4; 1.8], *p* = 0.0119) as well as vascularization score (1.3 [1; 2.6] versus 0.8 [0.3; 0.8], *p* = 0.0159). Bony ingrowth, fibrous interface width, and giant cell scores were higher in the FA compared to the NA tibial sections, but significance was not reached.

**Table 3:** Results quantifying the autograft osseointegration within the tibial bone tunnel at 12 weeks

	NA sections	FA sections	*p* value NA versus FA
Bone tunnel cross-sectional areas (µm^2^)			
Femur and tibia at 12 weeks	2638 [1547; 3537]	1675 [1341; 2121]	0.0615
Tibia at 12 weeks	2949 [1755; 3539]	1697 [1378; 2878]	0.2738
Tibia (*T*_0_ specimen)	1980	1870	–
Femur at 12 weeks	2326 [1481; 3694]	1668 [1165; 2010]	0.1548
Femur (*T*_0_ specimen)	2369	2012	–
Graft-to-bone score (tibia) (/15)	7.3 [4; 7.5]	9.3 [8.5; 12]	0.0040*
Fibrous interface width	2 [1.5; 2.3]	2.3 [2; 2.8]	0.123
Bony ingrowth	0.5 [0; 0.9]	1 [0.5; 2.3]	0.1032
Cellularity	1.3 [0.4; 1.8]	2 [1.9; 2.5]	0.0119*
Giant cells	2 [1.5; 2.5]	2.5 [2.4; 2.8]	0.0595
Vascularization	0.8 [0.3; 0.8]	1.3 [1; 2.6]	0.0159*

*Statistically significant

## Discussion

Anterior cruciate ligament (ACL) remnants preservation and reconstruction using autografts or synthetic scaffolds to promote ligament healing is a new emerging surgical strategy for the treatment of selected cases of ruptured ACL [10]. In order to develop an appropriate bio-implantable material allowing for ACL regeneration in vivo, a suitable small animal model that enables the assessment of ACL remnants fate and bone healing following remnants-sparing reconstructive procedures is required. To the authors’ knowledge, the model presented here is the first rat model of complete mid-substance torn ACL reconstruction in which the ACL remnants are preserved, and cortical screws are used as interference screws for direct autograft fixation, mimicking the surgical technique used in humans [41–43]. Flexor digitorum longus tendon, which was chosen in the present study, has already been used as an autograft in rats for anterior cruciate ligament reconstruction [44, 45], yet using other fixation techniques. Its use carries several advantages: (i) Biomechanical properties of the graft are satisfactory for this purpose; (ii) the sampling procedure is well tolerated by the animals; and (iii) this tendon is easy to identify as its manipulation involved a complete flexion of the digits (Fig. 1).

The fast return to weight-bearing in all rats in the present study proved that both surgeries, ACL transection and *rp*-ACLR, were well tolerated. The procedure-specific morbidity was low in both groups, even in the animals in which an autograft was harvested. The resulting temporary swelling and loss of digit flexion which was observed in these animals did not seem to impact activity level and weight-bearing. Surprisingly, in the IT group, at 12 weeks, lameness was not detected on visual gait analysis (further corroborated by patellar BMD ratios between left operated and right non-operated knees approximating 1) and Lachman tests were negative in most of the knees, despite a more severe amyotrophy, joint effusion, and cartilage degeneration in that group. Complete resolution of lameness by 12 weeks after ACL transection has indeed already been reported in large animal models experiments, based on visual gait and force platform analysis, and suspected to be due to prey-related behaviors, in which visible lameness could confer a survival disadvantage, and/or to muscular compensation of the knee instability, but the latter hypothesis is nevertheless not corroborated by the muscular amyotrophy observed in the IT group of the present study [46, 47]. Another explanation would be that both visual gait analysis and BMD evaluated 12 weeks postoperatively may be insufficient to characterize functional outcome in animals with isolated ACL section, which, as recently suggested, should be assessed in animal experiments using more clinically representative functional tests, but these are hardly applicable to animal models, for anatomical and animal compliancy reasons [16]. Nevertheless, gait analysis systems are widely used in the field of neuromuscular diseases research in rodents and could be of interest for this purpose [48].

In the coronal plane, 8/10 tibial and femoral bone tunnel aperture centers were anatomically positioned, with only 1% and 4% of deviation in median from the anatomical center of the ACL footprints, respectively, representing an absolute median deviation of less than 0.1 mm medially for the tibial and 0.3 mm laterally for the femoral offset in the coronal plane. In the sagittal plane, 6/10 tibial and femoral bone tunnel aperture centers were anatomically positioned, with 14 and 13% of deviation in median from the anatomic center of the ACL footprints, representing an absolute median deviation of less than 1 mm posteriorly for the tibial and 0.75 mm anteriorly for the femoral offset in this plane, which appear hardly improvable in such a small animal model. Moreover, even the most deviated bone tunnel apertures displayed some degree of overlapping with the corresponding ACL footprint. Variations in the bone tunnels positioning are well documented after  ACLR in humans but are very scarcely reported in animal experiments. Laurencin et al. reported variations of more than 4 mm in the anteroposterior position of their tibial tunnel apertures in a rabbit model of ACLR; they found that an anterior positioning of the tibial tunnel was associated with a higher likelihood of rupture after ACLR [49]. The only rat study that focused on the influence of bone tunnel aperture positioning was a cadaveric one in which the authors concluded that an anterior deviation of 1.5 mm from the isometric femoral footprint, who was assimilated in that study to the anatomical femoral footprint, resulted in an increase in graft tension at knee flexion angles above 45 degrees [50]. The lack of surgical guides designed for rat models was in part responsible for the variations observed in the present study in tunnels processing, given that femoral ACL footprints were not easily visible peroperatively, which is a well-known issue in people when ACL remnants are preserved [29]. Drilling the tunnels separately was a first step in improving their positioning, since the transtibial technique has been associated with less anatomical positioning [16, 42, 51]. Still, surgical custom-made drill guides systems should be developed to overcome this issue in the future, although it will remain hardly applicable to such a small animal model. Moreover, optimal femoral and tibial bone tunnel positions are still not defined in rats and might be different from the anatomic landmark, as is the “IDEAL” femoral tunnel aperture [42], which is also corroborated in the present study by the fact that a non-anatomic tunnel placement did not lead systematically to graft rupture, high OARSI scores, anterior instability, lameness, poor ACL remnants degeneration outcome, or bone tunnel widening, 12 weeks postoperatively. Based on these observations, bone tunnel aperture positioning was considered acceptable in this preliminary proof-of-concept animal model.

The model of the present study demonstrated its relevancy through the absence of spontaneous healing of the ACL 12 weeks after its transection without any subsequent stabilization. Macroscopic evaluation of the rat intraarticular knee structures is difficult and incomplete due to the small size of the joint in this model. Specifically, the posterior part of the autograft is difficult to visualize entirely. In the present study, MRI permitted to perform an accurate noninvasive evaluation of the ACL remnants and autograft integrity at 12 weeks, while preserving the articular tissues integrity for further histological processing. It also allowed the evaluation of its points of insertion, thus allowing the assessment of the anatomical or non-anatomical positioning of the bone tunnels (see dedicated section above). MRI is considered the modality of choice in humans for noninvasive ACL assessment and is widely used to evaluate reconstructed ACL [52]. In the current study, MRI provided evidence regarding the absence of ACL spontaneous healing at 12 weeks after mid-substance transection without reconstruction. It also permitted to visualize, in the AG group, the presence of ligamentous fibers spanning from femur to tibia and to assess the integrity of the *rp*-ACLR construct. However, the small size of the model did not allow to distinguish whether those fibers pertained to the autograft, to the ACL remnants, or to both. In addition, partial tears involving mild amount of tissue may not be visualized or differentiated from intact grafts by this imaging modality [52], although this may also be the case with macroscopic evaluation. MRI sensitivity and sensibility to detect graft failure was reported to be of 60 to 97.8 and 70 to 87%, respectively, in clinical settings [53, 54]. MRI is nevertheless an appealing tool in this field and deserves its place in experimental studies on ACL reconstruction in rats as it is in some rat models of post-traumatic osteoarthritis (OA) [55]. To overcome the limitations associated with its use, MRI findings can be supplemented by histological analysis [16].

Histology demonstrated a significant benefit of *rp*-ACLR over control transected ACLs regarding ACL degeneration at 12 weeks. Despite the absence of ACL remnants suturing, *rp*-ACLR induced only mild degenerative changes of the ACL remnants tissues, whereas severe degenerative changes were found in the ACL in knees in which transection was not combined with reconstruction [36]. These severe changes mostly consisted in a higher degree of inflammation, mucoid degeneration, chondroid metaplasia, and cystic changes compared to the reconstructed group. Collagen fibers disorganization has been reported to be the earliest and most prevalent change in the course of ACL degeneration, while cystic changes are relatively late events, and it was the only feature that did not differ significantly between both groups [36]. Histologic features of the ACL healing response after experimental transection, followed or not by surgery, are only scarcely described in the literature. Collagen fibers mild disorganization, decreased presence of voids and fat vacuoles, spindle-shaped fibroblasts, and neovascularization (assessed in the present study, respectively, through cystic changes, chondroid metaplasia, and inflammation criteria) were previously identified as healing response features in transected ACLs, 12 weeks after suture alone or combined with augmentation using small intestine submucosa [56]. Interestingly, neovascularization is sometimes considered as a healing, or as a degenerative pattern of ACL.

MRI and histology thus permitted in the present model to objectivate the absence of spontaneous healing 12 weeks after ACL transection without associated reconstruction. Some authors allocated the absence of spontaneous ACL healing to the gap formed between the ACL ends and subsequent interposition of synovial fluid, which impair the possibility of a guided regeneration process [5]. Indeed, healing of ACL remnants has been previously observed on histology 12 weeks after surgery in a goat model of mid-substance transection followed by locking suture, alone or combined with small intestine submucosa [56]. Some authors suggested spontaneous healing capacities of the ruptured ACL when in contact with its surrounding tissues [57]. These mechanisms could explain the mild degenerative changes observed histologically on ACL remnants when reconstructed with the autograft, even in the absence of ACL remnants repair using sutures, which was not performed in our study because of the small size of the ACL in rats.

A strong trend was found for FA to be smaller than NA bone tunnel areas (*p* = 0.06). Median FA areas at 12 weeks being inferior to corresponding FA areas on T_0_ specimen, a narrowing of the FA part of the bone tunnels following surgery was suspected. Contrary to FA areas, NA areas of the T_0_ specimen suggested that the majority of specimens exhibited an enlargement of the NA part of the bone tunnels at 12 weeks. Histology findings corroborated those results as graft-to-bone scores for osseointegration of the AG were significantly increased in FA sections compared to NA sections, reflecting an increased bone healing at this location. Those discrepancies between NA and FA sections may be explained by biological and mechanical variations between the parts of bone tunnels located near the articulation and far from it. Indeed, enlargement of NA bone tunnels is a well-known fact, experimentally and clinically [58]. Synovial influx within the bone tunnels and mechanical stress provided by the graft mobilization over the edges of the tunnel articular outlet were both contemplated as explanative mechanisms. Some authors suggested that remnants-preserving techniques may prevent NA bone tunnel enlargement as the presence of ACL remnants would limit the synovial influx within bone tunnels [59]. Similar hypotheses were made for direct fixation of the graft using interference screws [60] positioned near the joint line (“from the inside”) [41]. In the present study, fixation was provided by screws inserted from the outside of the bone tunnels and not positioned directly at the joint line [41], which may have acted favorably on bone formation in FA sections, providing a biomechanically favorable environment, and filling the bone tunnels so as to prevent synovial fluid from penetrating; this effect was, however, not present at NA sections. Indeed, the use of cortical screws as interference screws did not permit their insertion from the inside of the joint because of the presence of a screw head that cannot be placed in the intraarticular compartment.

To the authors’ point of view, fixation using cortical screws was easy and fast to perform, and it might have provided more secure fixation than periosteal and soft tissue nearby sutures that are commonly used in ACL standard reconstruction rat models [16]. Moreover, soft tissue release associated with these techniques may induce mechanical strength loosening over time, which is detrimental to graft tension and thus to the overall lead experiment [16]. Direct fixation using cortical screws overcomes this issue and, in addition, limits swing range and elongation of the graft since those features increase with the distance from the fixing point [58]. Most rabbit models applied interference screws on the femur side but not on the tibial side because of insufficient trabecular bone at this location to hold the screw [16]. In the present rat model, peroperative anchorage of both femoral and tibial screws was good in all rats, and no screw migration or graft loosening or sliding has been observed at 12 weeks, suggesting that cortical screw is also applicable on the tibial side in rats. Moreover, both tibial and femoral tunnels were surrounded by cortico-cancellous bone at 12 weeks, as documented through both histology and micro-CT analysis. The use of cortical screws inserted in a semi-anatomical manner was thus efficient for graft anchorage in both femoral and tibial tunnels.

Our multimodal evaluation of OA did not reveal significant differences between the autograft reconstructed group and the transected ACL group on total RAKSS scores. However, when looking in a detailed manner at this scoring system, the reconstructed group had significantly less degenerative cartilage changes and joint effusion compared to the transected group, corroborating the measurements of synovial membranes thickness that were significantly thinner in the reconstructed group. Evidence is still lacking in people regarding the superiority of ACL reconstructive surgery over conservative management on post-traumatic OA [2, 61]. Some studies even suggested a worse OA long-term prognosis for operated ACL-deficient knees compared to knees treated conservatively [5]. Experimental studies in animals demonstrated that ACL reconstruction did not protect cartilages from degenerative changes [62]. Explanative hypotheses rely on the fact that ACL reconstructive surgery does not restore normal joint mechanics and may induce prolonged joint inflammation deleterious to tissues healing, or that damages caused to the joint tissue are not readily reversible through joint stabilization [2]. Another explanation for this may be the short time period at which evaluation in animal models is performed. Short-time outcome may indeed not reflect longtime outcome which is an important issue in OA as it increases significantly with time [63]. In the present rat model, *rp*-ACLR did reduce the development of joint effusion and cartilage damages compared to transected ACL group, corroborating the findings of a study in a porcine model in which ACL augmentation, bio-enhanced with autologous blood containing platelets, provided protection from cartilage damages [64], highlighting the benefits of remnants-preserving ACL surgeries over conventional ACL reconstruction.

Some limitations should be noted in this study. Firstly, biomechanical studies were not conducted to assess the stiffness of the ACL/autograft constructs. Conclusions could therefore only be drawn on a clinical and a biological point of view. Secondly, the small number of animals may have induced a lack of statistical power. Nevertheless, significant differences or strong trends emerged from the comparison of both groups with only 5 specimens per group, attesting for the relevance of this model and optimizing the 3Rs (Replace, Reduce, Refine). Another limitation of our study was that the benefits of *rp*-ACLR over standard ACLR could not be demonstrated in the present model because comparison between remnants preservation and remnants excision was not made. Yet, because the present model was developed to serve as positive control for further evaluation of synthetic ligaments developed to be used instead of the autograft in remnants-preserving reconstruction strategies, the design of our study did not include a group in which the anterior cruciate ligament was completely excised.

## Conclusion

To the authors’ knowledge, the present rat model establishes for the first time a new surgical model of *rp-*ACLR with a tendinous autograft after mid-substance tear. The model was associated with a low morbidity, which allowed the study on small number of animals per group, in agreement with the 3R rule. We demonstrated its relevancy through the absence of spontaneous healing of the ACL 12 weeks after its isolated transection (negative controls) and a significant improvement in the ACL remnants degenerative status after mid-substance transection, when remnants preservation was associated with reconstruction using a tendinous autograft (positive controls) with less cartilage degenerative changes, joint effusion, and synovitis. This cost-effective and clinically relevant animal model can be used for the study of bio-implantable materials designed for *rp-*ACLR. It also brings the possibility to perform mechanistic studies such as bioluminescence imaging which could be a useful tool in the field of tissue engineering.

## Data Availability

The datasets used and/or analyzed during the current study are available within the article. Additional data are available from the corresponding author, EM, on reasonable request.

## Abbreviations

ACL: Anterior cruciate ligament
AG: Autograft
BMD: Bone mineral density
FA: Far from articulation
FOV: Field of view
IP: Intra-peritoneally
IT: Isolated transection
MRI: Magnetic resonance imaging
NA: Near articulation
NEX: Number of experiments
OA: Osteoarthritis
*rp*-ALCR: Remnants-preserving anterior cruciate ligament reconstruction
SC: Subcutaneously
Tacq: Acquisition time
TE: Echo time
TR: Repetition time
